# Digital Gene Expression Profiling by 5′-End Sequencing of cDNAs during Reprogramming in the Moss *Physcomitrella patens*

**DOI:** 10.1371/journal.pone.0036471

**Published:** 2012-05-04

**Authors:** Tomoaki Nishiyama, Kaori Miyawaki, Masumi Ohshima, Kari Thompson, Akitomo Nagashima, Mitsuyasu Hasebe, Tetsuya Kurata

**Affiliations:** 1 Exploratory Research for Advanced Technology, Japan Science and Technology Agency, Okazaki, Japan; 2 Advanced Science Research Center, Kanazawa University, Kanazawa, Japan; 3 Division of Evolutionary Biology, National Institute for Basic Biology, Okazaki, Japan; 4 School of Life Science, The Graduate University for Advanced Studies, Okazaki, Japan; Oregon State University, United States of America

## Abstract

Stem cells self-renew and repeatedly produce differentiated cells during development and growth. The differentiated cells can be converted into stem cells in some metazoans and land plants with appropriate treatments. After leaves of the moss *Physcomitrella patens* are excised, leaf cells reenter the cell cycle and commence tip growth, which is characteristic of stem cells called chloronema apical cells. To understand the underlying molecular mechanisms, a digital gene expression profiling method using mRNA 5′-end tags (5′-DGE) was established. The 5′-DGE method produced reproducible data with a dynamic range of four orders that correlated well with qRT-PCR measurements. After the excision of leaves, the expression levels of 11% of the transcripts changed significantly within 6 h. Genes involved in stress responses and proteolysis were induced and those involved in metabolism, including photosynthesis, were reduced. The later processes of reprogramming involved photosynthesis recovery and higher macromolecule biosynthesis, including of RNA and proteins. Auxin and cytokinin signaling pathways, which are activated during stem cell formation via callus in flowering plants, are also activated during reprogramming in *P. patens*, although no exogenous phytohormone is applied in the moss system, suggesting that an intrinsic phytohormone regulatory system may be used in the moss.

## Introduction

Differentiated cells can be reprogrammed to become stem cells in some mammals and land plants with appropriate inductive treatments [reviewed in 1,2]. In flowering plants, exogenous application of the phytohormones auxin and cytokinin to the culture medium induces proliferation of explanted differentiated cells. The proliferated cells form either somatic embryos or a mass of cells termed callus [3]. Somatic embryos, like regular embryos, form shoot and root meristems, in which stem cells are initiated. Callus cells form shoot or root meristems depending on the ratio of exogenous auxin and cytokinin in the culture medium [4].

**Figure 1:**
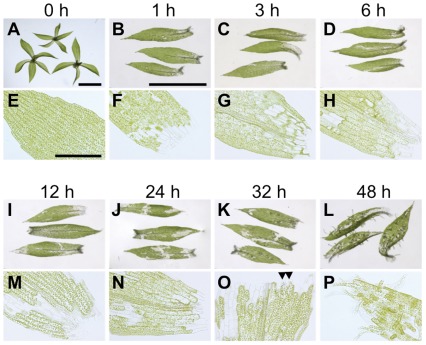
Reprogramming from leaf cells to chloronema apical cells. (**A**) Upper parts of gametophores. (**B–D**, **F–P**) Dissected leaves after indicated time. (**E**) Intact leaf. Bars in **A**: 1 mm; **B**: 1 mm for **B–D** and **I–L**; **E**: 0.2 mm for **E–H** and **M–P**.

In mosses, which diverged from flowering plants in the Silurian [5], vegetative cells do not need exogenous application of phytohormones for their reprogramming into stem cells [6]. The feasibility of gene targeting with high homologous recombination rates [7], [8] and the availability of genome information [9] make the moss *Physcomitrella patens* suitable for stem cell studies. When a leaf of a *P. patens* gametophore, a haploid shoot, is dissected and incubated in culture medium without exogenous phytohormones, leaf cells facing the dissected cells start to protrude with tip growth within a few days [10]. These protruded cells have characteristics of chloronema apical cells, which are a type of stem cell in *P. patens*. In regular development, chloronema apical cells are formed after spore germination and they self-renew and repeatedly produce chloronema cells. As chloronema apical cells, the reprogrammed leaf cells reenter the cell cycle, self-renew, and produce cells indistinguishable from chloronema cells (Figure 1).

Such direct changes of differentiated cells into stem cells have not been observed in flowering plants, in which somatic embryo or callus is always formed before the formation of stem cells. Molecular mechanisms, especially of the changes in gene expression during callus formation and subsequent meristem initiation, have been well studied in several flowering plants and conspicuous changes in transcripts involved in auxin and cytokinin signaling and downstream transcription factors have been observed [11], [12], [13], [14], [15], [16]. On the other hand, transcriptome profiles during the reprogramming process of mosses have not been studied. Thus, the differences and similarities in expression profiles during the direct change into stem cells in the moss compared to callus and meristem formation in flowering plants are unknown.

Massive parallel sequencing technology has enabled measurements of the expression levels of transcripts with a large dynamic range by sequencing cDNA. RNA-seq is a method involving sequencing short reads covering the entire region of a transcript [17], and TSS-seq specifically detects transcriptional start sites by employing the oligo-capping method [18], [19]. RNA-seq data are usually normalized for the length of the transcript as RPKM [20]. The stochastic error is roughly proportional to the square root of the number of tags but not to that of RPKM, and having more tags for longer transcripts necessitates more total reads to achieve a given level of stochastic error for shorter transcripts.

5′-end sequencing with oligo-capping [18] requires multiple enzymatic reaction steps to remove the endogenous cap structure and ligate a synthetic oligonucleotide before converting the transcript to cDNA. Plessy et al. recently reported a new method called nanoCAGE [21], which utilizes template switching [22], [23] to capture transcriptional start sites (TSSs) from small amounts of RNA [21]. Although nanoCAGE is useful to detect a wide variety of TSSs in a genome-wide manner [21], its quantitative nature with regard to mRNA accumulation is unclear.

Here, to analyze expression profiles of *P. patens*, we employed the template-switching reaction and established a simpler method for detecting the 5′-end of mRNA and obtaining a quantitative digital measure of transcripts using a SOLiD parallel DNA sequencer (Life Technologies). Transcriptome profiles during moss stem cell formation were investigated from early time points through the time at which reprogrammed cells protrude and divide. The expression profiles showed that transcripts sequentially changed by 6 h after dissection, which is earlier than the detection of tip growth and cytokinesis at approximately 24 h after dissection. Comparison of the profiles to those of stem cell formation via callus in flowering plants revealed that phytohormone-induced genes in flowering plants are also activated in *P. patens*, suggesting that the moss utilizes intrinsic phytohormone regulatory systems for reprogramming. We further found that members of the same gene families are dynamically changed during the reprogramming process in flowering plants and the moss, suggesting that common factors are involved in the regulation of reprogramming in land plants in general.

## Results

### Construction of a 5′-digital Gene Expression (5′-DGE) Library Using the Template-switching Method

**Figure 2:**
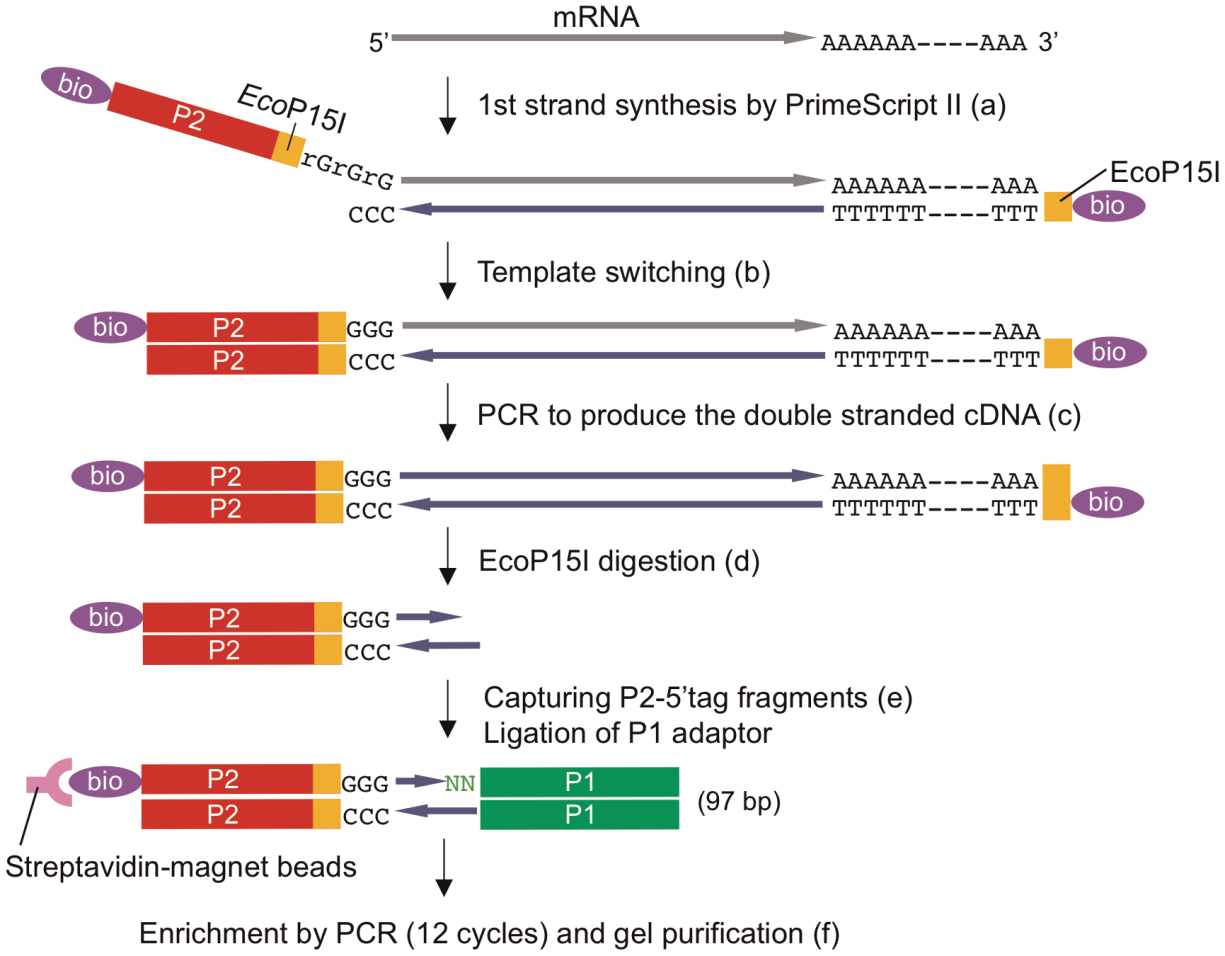
Workflow for 5′-DGE library preparation. (a) 1st strand cDNA is synthesized from mRNA. (b) At the 5′ end of the mRNA, cDNA synthesis continues onto the DNA/RNA chimeric oligonucleotide. (c) Three-cycle PCR is performed to produce the double-stranded cDNA. (d) the double-strand cDNA fragments are digested by EcoP15I. (e) Digested P2-attached 5′ tag fragments are captured by streptavidin-magnet beads and ligated with P1 adapter. (f) 5′-DGE library is amplified, and 97 bp fragments are purified after PAGE.

The template-switching method [23] was employed for the construction of a 5′-SAGE-like library for SOLiD sequencing. Figure 2 shows the workflow for 5′-digital gene expression (5′-DGE) library construction. The reverse transcription started from biotin TEG-labeled EcoP15I-dT_20_ at the polyA tail toward the 5′ end of the mRNA. PrimeScript II reverse transcriptase was selected for high efficiency in reverse transcription and template switching. The reverse transcriptase added a few nucleotides (dC) at the 3′ end of synthesized cDNA. After switching the template, cDNA synthesis continued to the biotin TEG-labeled DNA/RNA oligonucleotide containing an EcoP15I site (BioTEG-P2EcoP15I-rGx3). Double stranded cDNAs were amplified via 3 cycles of PCR and digested by EcoP15I to produce DNA fragments consisting of P2EcoP15I and 25 bp of cDNA with a two-base 5′ overhang. Digested fragments were captured with streptavidin beads and ligated with P1 adaptors containing two-base overhangs. The resulting 25 bp 5′ cDNA fragments sandwiched by P1 and P2EcoP15I adaptors were amplified by 12 additional cycles of PCR for SOLiD 25 bp sequencing. The sequencing started at P1 to avoid having all templates contain the same EcoP15I sequence during the early cycles. Thus, the sequence starts 25 bp downstream of 5′ end of the RNA toward the 5′ start site. When unmodified EcoP15I-dT_20_ was used for reverse transcription, byproducts containing the EcoP15I-dT_20_ sequence were observed. These byproducts were suppressed by introducing the 5′-end biotin-TEG modification to EcoP15I-dT_20_.

### Massively Parallel Sequencing of 5′-DGE Libraries

Before obtaining time-course data, to confirm the stability and reproducibility of the method, two independent 5′-DGE libraries (technical duplicates; test1 and test2) were constructed from the same total RNA sample isolated from a mixture of gametophores, protonemata and rhizoids. The libraries were sequenced for 25 bp on SOLiD2 and 3 systems (Life Technologies). Mapping statistics of each library are summarized in Table S1 The proportion of mappable tags was about half with SOLiD2 and increased to two-thirds with SOLiD3, which are comparable to previous reports in a different system [24]. The unmapped reads may contain splice junctions and reads having more than two errors. The proportion of low-information reads with fewer than four non-0 color bases ranged from 0.3 to 5% of the mappable tags and may represent differences in library preparation or instability of the instrument. The percentage of mappable tags that were uniquely mapped ranged from 82% to 90%. Excluding the low-information reads, the percentage ranged 85% to 90%. The proportion of uniquely mapped tags increased during the reprogramming process, implying that the expression of non-repetitive genes increased in comparison to repetitive elements (Table S1).

### Calculation of Expression Level per Gene

**Figure 3:**
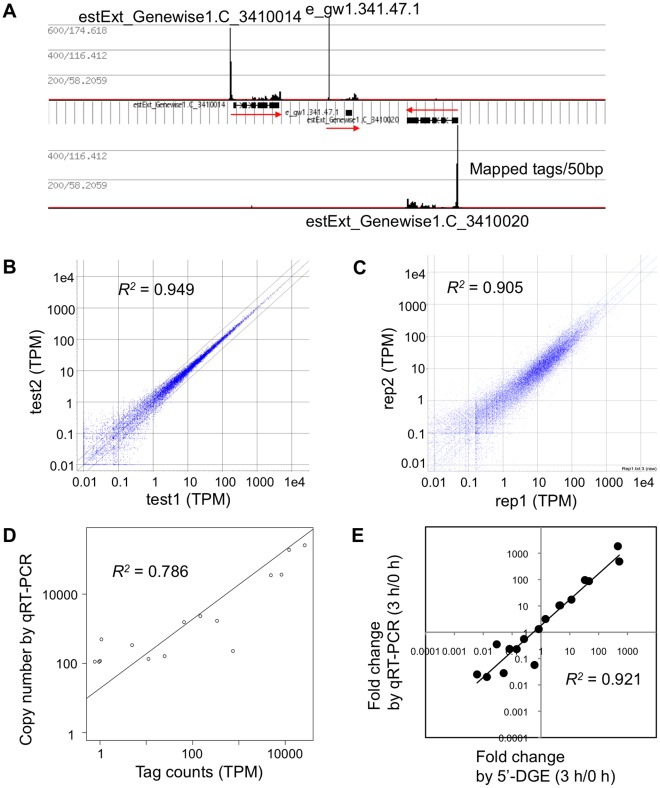
Qualitative and quantitative nature of the 5'-DGE tags. (**A**) Tags mapped to reverse and forward strands, representing forward- and reverse-strand transcripts, are shown in the upper panel and lower panel, respectively. Exons and introns of three gene models are shown by black boxes and horizontal lines. The values given on the left define the level of raw tag/normalized tag (genome average = 1). (**B**) Scatter plot of technical replicates (test1 and test2) in tags per million (TPM). (**C**) Scatter plot of biological replicates (rep1 and rep2). (**D**) Correlation between tag counts and the copy number per 0.1 ng polyA RNA measured by qRT-PCR (test1 sample). (**E**) Correlation of fold changes measured by SOLiD-DGE tags and by qRT-PCR. The ratios between 0 and 3 h samples were compared.



The tag distributions on several genes were investigated. It was frequently observed that a sharp peak existed 5′ of the annotated gene model and lower levels of tags were distributed over the exons (Figure 3A). The 5′ peak corresponded well to the 5′ proximal end of the 5′ EST of full-length cDNA libraries constructed with the cap-trapper method [25]. The tag in the peak was thus considered to originate from the capped mRNA of the corresponding gene. The tags on the exon could be derived from truncated mRNA, from a template-switch event occurring before the 5′ end was reached during reverse transcription, or from alternative transcription start sites. Only 7,179 out of 35,937 had 5′ UTR annotation and 44% of tags were out of the region annotated as transcripts. To count the number of tags for a gene including the untranslated and coding regions, we defined a region for each gene that is wider than the annotated gene model, because the gene model often lacks annotation for untranslated regions. The length of the extension was defined as 2 kb for the 5′ end and 1 kb for the 3′ end. In cases in which two genes with the same direction are present within a short distance, the intervening region was divided for the two genes so that the expansion did not create an overlap. While 56% of mapped tags was on the originally annotated region, 94% was on the expanded region in the test1 sample. The regions comprise 180 Mbp or 38% of the genome after the expansion. The number of tags mapped to the region was counted and divided by the number of tags mapped to the whole reference sequence to obtain the tags per million (TPM) value.

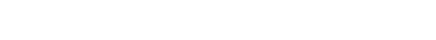
.

At this time, the tags that match to multiple locations with the same number of mismatches were treated as ambiguous and fraction numbers (1/*n* if it match *n* locations) were added to each location. When we discard all ambiguously mapped tags and count only uniquely mapped tags, a similar number was obtained for most genes (identical for 10,326 genes), but 956 genes having more than 1 TPM showed large drop to less than half as shown Figure S1.

The relationship of the length of exon and the TPM value was investigated in the test1 sample to examine if the tags from long transcripts are actually captured by this method, involving reverse transcription from polyA, we investigated (Figure S2). We found that longer genes do have similar number or more tags compared to shorter genes, although the levels vary among genes.

Because EcoP15I recognition sequence may perturb the data as the enzyme was used in the library construction, we counted the cumulative tag counts around forward (CAGCAG) and reverse (CTGCTG) sequences (Figure S3). The results suggested that the reverse site enhanced tags 5′ to the site and suppressed for 20 bp region 3′ to the site by a factor of about 3 compared to more 3′. In the forward site, there was a slight trend that more tags were observed 3′ to the site, and a sharp peak existed at position 32 (0 is the first C of CAGCAG). Thus presence of the EcoP15I sequence may perturb the tag count of the mRNA, but does not totally diminish the count at surrounding positions.

We found a high correlation between the technical duplicate data for the 5′-DGE libraries, indicating that the 5′-DGE system is highly reproducible (Figure 3B, Pearson’s correlation coefficient, *R^2^* = 0.949). 17,170 and 17,372 genes had more than 1 TPM in the technical duplicates respectively and 16,808 were more than 1 TPM in both. 24,915 and 24,943 genes had at least 1 tag in the technical duplicates respectively, and 23,179 genes were detected in both. If we look at only the originally annotated region without expansion, only 13,449 genes are more than 1 TPM and 21,036 had at least 1 tag. For biological replicates, high correlation was also observed (Figure 3C, *R* *^2^* = 0.905,).

The amounts of mRNAs measured by qRT-PCR and tag counts were compared to assess the detection limit and dynamic range of the 5′-DGE method. Figure 3D shows good correlation between the two methods at a dynamic range of four orders (*R^2^* = 0.786). For quantitative analysis of gene expression, the detection limit was around 1 TPM, which corresponds to approximately 100 copies in 0.1 ng polyA RNA (Figure 3D). Technical duplicates showed only small deviation at TPM values greater than one (Figure 3B). The expected stochastic error at 1 TPM is 20–30% with the number of tags sequenced.

### Gene Expression Profiling during Reprogramming from Leaf Cells into Chloronema Apical Cells

The 5′-DGE method was employed to analyze gene expression patterns during the reprogramming of leaf cells into chloronema apical cells in *P. patens* (Figure 1). Chloronema apical cells are formed from approximately 30% of leaf cells in dissected leaves by 48 h and they continue to form chloronema cells. For 5′-DGE library preparation, upper parts of leafy gametophores without rhizoids and protonema were dissected with a homegenizer as described in Ishikawa et al., 2011 [10]. Leaves were collected 1, 3, 6, 12, and 24 h after dissection and used to make 5′-DGE libraries for sequencing. As a 0 h control sample, harvested gametophores were immediately frozen for RNA extraction.

Obtained sequence tags were mapped to the *P. patens* reference genome and the number of mapped sequence tags for each gene model including both 5′ and 3′ putative untranslated regions was counted to calculate the TPM value. Biological triplicates for the dissected leaves at each time-point were subjected to sequencing and analyzed with GeneSpring ver. 7. Pearson correlation coefficient *R* ^2^ values ranged from 0.879 to 0.956 in the triplicates (Figure S4).

### Evaluation of Fold-changes by 5′-DGE and by qRT-PCR

The amounts of transcripts of sixteen genes were analyzed with qRT-PCR using RNA samples used for the 5′-DGE analysis to validate the fold-change values calculated with 5′-DGE tag frequencies ([*TPM* in sample 1]/[*TPM* in sample 2]). Fold-changes between 0 h and 3 h RNA samples were highly correlated between 5′-DGE tags and qRT-PCR results (*R^2^* = 0.921) (Figure 3E).

### Unsupervised Characterization of Gene Expression Changes during Reprogramming

**Figure 4:**
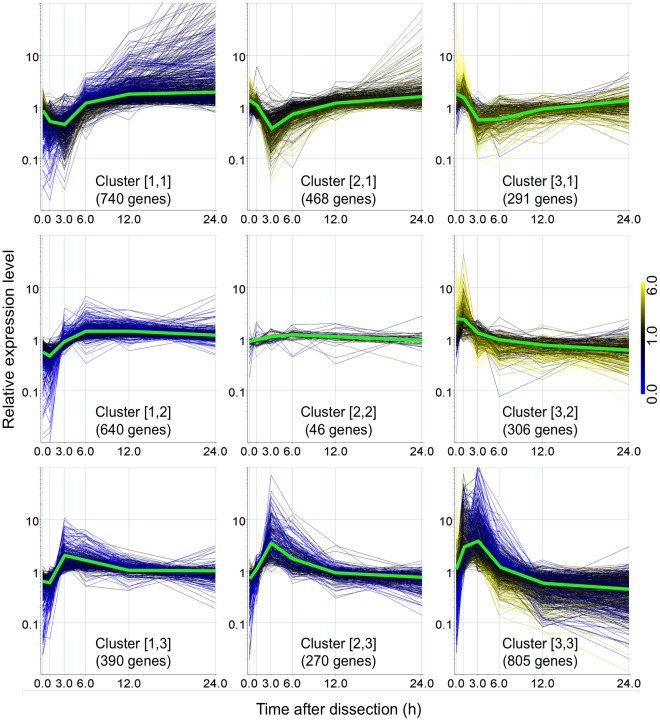
SOM-based clustering analysis for time-course samples. 3,956 genes with significant expression changes were clustered into 3 by 3 groups with a SOM algorithm. Relative expression changes for each gene are shown in the corresponding cluster. Green lines in individual clusters indicate the average calculated from the relative expression levels of the genes. Line color corresponds to the relative expression level at 0 h (scale to the right).

Genes with significant differences in the number of 5′-DGE tags during the reprogramming process were selected by one-way ANOVA with a 5% false discovery rate (FDR) and 3,956 genes passed the filter as differentially expressed. These genes were clustered into 3 by 3 gene sets using the self-organizing map (SOM) algorithm [26] (Figure 4). As judged from an overview of the expression pattern clusters (Figure 4), drastic changes of gene expression occurred by 6 h after dissection and prior to the acquisition of tip growth at 24 h after dissection. Cluster [2,2] contains genes with only small fold-changes, although the changes were statistically significant according to ANOVA tests.

Gene ontology (GO) term enrichment analysis was employed to characterize the clusters and the BiNGO tool [27] was used to investigate statistically overrepresented gene sets defined by association with a GO term in each cluster (Table S2).

#### Early transient response within 6 h

Clusters [3,3] contained genes whose transcripts accumulated predominantly at 1 or 3 h, respectively. These clusters included genes related to stress responses. In cluster [3,3], a significant overrepresentation of protein kinases was observed (Table S3). Among these kinases are genes coding for proteins similar to CERK1 (CHITIN ELICITOR RECEPTOR KINASE 1), and calcium -dependent kinases, which are involved in stress responses in flowering plants [28], [29].

Clusters [1,1], [2], [1], and [3], [1] are composed of genes transiently down-regulated mostly at 3 h after dissection. Genes involved in photosynthesis were overrepresented in clusters [2], [1] and [3], [1] (Table S2), and “biosynthetic process” and “cell wall organization” were overrepresented in cluster [1,1]. After the reduction of expression of these genes at 3 h, these transcripts increased gradually by 24 h.

#### Expression changes in processes occurring after 6 h

Cluster [1], [2] includes genes with a low expression level at 1 h, but up-regulated after 6 h. Genes involved in RNA processing and translation were overrepresented in this cluster (Table S2). Genes labeled “mitochondrial part” and cell-wall related genes were overrepresented in this cluster.

### Expression of Orthologs Involved in Phytohormone Signaling, Callus Induction, and Stem Cell Formation in *A. thaliana*

**Figure 5:**
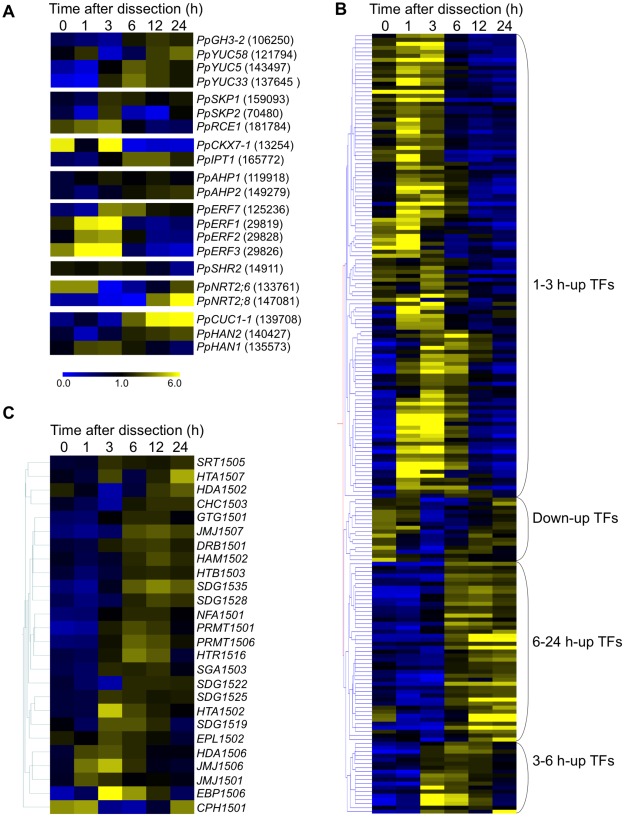
Expression patterns of transcription factors, epigenetic-related and reprogramming-related genes. (**A**) Heat map representation of expression patterns during reprogramming for reprogramming-related genes. Numbers in parentheses indicate JGI protein identifiers. (**B**) Expression patterns of transcription factor genes sorted according to gene expression pattern tree. Four distinct clusters are identified to the left. (**C**) Expression patterns of epigenetic-related genes.

We next focused on expression dynamics of *P. patens* genes homologous to *A. thaliana* genes involved in auxin and cytokinin signaling, both of which function in callus and somatic embryo formation in angiosperms [12], [15]. Seventy-three genes were analyzed and eleven genes showed significant changes in transcript accumulation. Transcripts of homologs (ids:143497 and 137645) of the auxin biosynthetic gene *YUCCA* [30] decreased 1 h after dissection but increased by 3 h and was maximal at 6 h. Another *YUCCA* homolog (id:121794) had biphasic expression pattern; up-regulated at 1 h and 6 h (Figure 5A). *P. patens* auxin conjugation enzyme PpGH3-2 [31] functions to inactivate auxin. *PpGH3-2* (id: 106250) transcripts decreased until 3 h after dissection and then increased until 6 h (Figure 5A).

Among auxin signaling-related genes, a *RCE1* homolog *PpRCE1* (id: 181784) was induced by 1 h and down-regulated after 6 h. The expression of a homolog *PpSKP1 and PpSKP2* (ids: 159093 and 70480) of another auxin signaling gene *SKP1* decreased at 1 h but was induced by 3 h and down-regulated at 6 h.

Among cytokinin signaling factor genes, histidine-containing phosphotransfer protein gene *PpAHP1* (id: 119918) was induced by 3 h and *PpAHP2* (id: 149279) was transiently repressed at 1 h. For cytokinin metabolism, *PpIPT1* (id: 165722), a moss homolog of cytokinin biosynthetic isopentenyltransferase gene (*IPT*), was induced at 3 h. The expression of a homolog *PpCKX7* (id: 13254) of the cytokinin oxidase/dehydrogenase 7 gene (*CKX7)* decreased at 1 h but was induced by 3 h and down-regulated at 6 h.

We also analyzed homologs of *A. thaliana* genes that function in the initiation and maintenance of stem cells (Table S4). *P. patens* forms a unicellular stem cell different from the multicellular stem cells in a flowering plant meristem. A *SHR* homolog *PpSHR2* (id: 14911) gradually decreased during the process. In contrast to the expression pattern of *PpSHR2*, a *CUC1* homolog *PpCUC1-7* (id: 139708) was gradually up-regulated (Figure 5A). *HAN* homolog *PpHAN1* (id: 135573) was transiently up-regulated with a peak at 1 h (Figure 5A). The expression of other HAN homolog *PpHAN2* (id: 135573) decreased at 1 h but was induced by 3 h The gradual reduction in *SHR* ortholog expression implies that the *SHR* ortholog may function in leaves but not in chloronemata while *CUC1* homolog may have a role in chloronemata. The *A. thaliana HAN* promoter is activated by H_2_O_2_. The early induction of its moss homolog may reflect conservation of the stress responsive regulation of *HANs*. *Pp NRT2;6* and *Pp NRT2;8* (ids: 133761 and 147081), homologs of *A. thaliana NRT2.1* which regulates the lateral root initiation in response with nutritional cues [32], were transiently down-regulated at 3 h then up-regulated, and up-regulated after 6 h, respectively.

The reprogramming process is triggered by dissection of leaves, thus action of the wound-related phytohormones jasmonic acid [33] and ethylene [34] may also play important roles. *A. thaliana ERF1* encode transcription factors involved in ethylene signaling. Four *ERF1* homologs were induced during the early phase of reprogramming (1–3 h) in *P. patens* (Figure 5A).

### Transcription Factors and Epigenetic Regulation-related Genes that Showed Significant Change of Expression during Reprogramming

In embryonic stem (ES) cells, pluripotency is controlled by epigenetic factors, including chromatin structure, DNA methylation, and miRNA, in concert with transcription factors [35]. These factors potentially regulate multiple genes and may be efficiently used during reprogramming in which many genes changes their expression. These factors were investigated in the 5′-DGE profiles during the reprogramming. A gene expression pattern tree of transcription factors during the reprogramming was constructed after selecting genes that showed an expression level change by 1-way ANOVA (Table S5). We could find four different patterns of up-regulation of these transcription factor genes (Figure 5B). Three clusters were induced at 1 to 3 h, 3 to 6 h, or 6 to 24 h after dissection. The other one had a negative peak at 3 to 6 h. Two ERF-type transcription factor genes (ids: 126548 and 29828) and a TIFFY/ZIM gene (id: 17332) up-regulated at 1 h are reported to be induced by salt stress [36].

Epigenetic regulation-related factors are involved in many biological processes. To investigate whether there were epigenetic factors showing expression change, genes encoding histone H2A, histone H2A variants, histone modification enzymes, DNA methylase, small-RNA biosynthesis-related and chromatin-remodeling factors (Table S6) were selected for the analysis, and a gene expression pattern tree is shown in Figure 5C. Different classes of genes for epigenetic regulation-related factors were up-regulated with peaks at 1, 3, 6, or 12 h in gene-dependent manner. Histone deacetylase *HDA1502* and SMARTD/SWP73/RSC6 group gene *CHC1503* were transiently down-regulated with the negative peak at 3 h. Transcripts of condensin-subunit H *CPH1501* gene decreased at 3 h then gradually increased until 24 h.

## Discussion

### Establishment of a Method for Quantitative Digital Gene Expression Analysis via Massively Parallel Sequencing

We established a novel quantitative DGE method by using template-switching based 5′-end capture and a massively parallel sequencing platform. The template-switching procedure has advantages for library construction: the usual 5′ SAGE-like approaches require a few hundred micrograms of total RNA [18], [19], whereas 4 µg is enough to construct a 5′-DGE library using our protocol. Furthermore, 5′-DGE library construction is simpler; there is no requirement for enzymatic treatments for cap structure removal and linker ligation to 5′ ends of RNAs. Although the adapter sequence was designed for fragment sequence runs with the SOLiD system, the sequence can be changed to facilitate barcode sequencing or to be used with other platforms.

Whereas nanoCAGE [21], which is also based on template switching, was designed to capture genome-wide transcriptional start sites, the 5′-DGE method reported here is demonstrated to produce quantitative genome-wide expression data that displays a high correlation with quantification by qRT-PCR and even better correlation in terms of fold-change. An advantage of the nanoCAGE method is that it is applicable to very small amounts of RNA, samples of as little as 10 ng can be used by employing semisuppressive PCR before EcoP15I digestion, whereas the method presented here uses a minimal number of PCR cycles to diminish the skew potentially introduced by PCR. The data from nanoCAGE includes non-polyA RNA species because random primers are included in the reverse transcription reaction, whereas the 5′-DGE method specifically selects polyadenylated RNA. Dependency of template switching on the presence of cap structure is controversial [21], [24] and it may vary based on the particular enzyme and conditions used for the reverse transcription. Whereas the developers of the nanoCAGE method observed enrichment of capped RNA by the template-switch reaction, whether capped RNA is enriched in the 5′-DGE method is unclear, as we observed tags on exons other than those that are proximal to the 5′ end of the transcript.

While we simply counted all tags on the region, which will give estimates similar to what would be measured by qPCR of 3′ UTR, counting tags only in 5′ UTR might be sensible as estimator of translatable intact RNA molecules. Such counting after more thorough annotation may be useful for study of RNA dynamics.

In contrast to microarray-based techniques, deep sequencing approaches do not require probe design. This advantage is more prominent in organisms other than typical model species, in which EST data are not abundant. To obtain a good microarray for expression analysis with small amount of RNA, probes are recommended to be designed on 3′ UTR, which is virtually impossible to infer *in silico* instead of from EST data. The calculation of expression levels as TPM values is simple and the TPM values have good correlation with the expression levels measured by qRT-PCR without needing to be divided by the length of the mRNA, which is often not precisely known in non-typical model organisms. Although sequence specific biases inherent of the EcoP15I exist, higher correlation in relative quantification in terms of fold-change can be obtained (*R^2^* = 0.921). The 5′-DGE method provided a dynamic range spanning four orders and a lower limit at 100 copies/0.1 ng polyA RNA in a quantitative manner. The stochastic error can be further reduced to improve the detection limit by sequencing more tags. Further deep statistical investigation on the nature of the tags may reveal a better treatment of the data. Since the 5′-DGE tag distributes over the exons and has a strong peak at the 5′ proximal region, the data may be utilized to improve the gene models in the future.

### Sequential Changes in Gene Expression during Reprogramming from *P. patens* Leaf Cells to Chloronema Apical Cells

5′-DGE analysis was applied to generate gene expression profiles during the reprogramming from *P. patens* leaf cells to chloronema apical cells with stem cell characteristics. In this profiling, the biological samples contained both reprogrammed and non-reprogrammed cells, which might result in low signal/noise ratio. To evaluate 5′-DGE method for time course profiling, the expression of a known cell cycle marker was investigated in our 5′-DGE data. *PpCYCD;1*, *Physcomitrella CYCLIN D;1*, is up-regulated at 12 h, and promoter-reporter experiment showed that *PpCYCD;1* was accumulated only at cut edge region [10]. Similar up-regulation during reprogramming was observed in 5′-DGE time course data (Figure S5).

Statistical analysis revealed that one tenth of all genes (3,956 out of 35,937 gene models) were differentially expressed at one or more time points from 0 to 24 h. SOM clustering based on expression patterns divided these differentially expressed genes into nine clusters (Figure 4). Large-scale expression changes occurred in a specific time window, between 0 and 6 h, as shown in Figure 4. Similar massive changes for gene expression were observed at a callus-inducing step at 2 or 3 days after placement of *Arabidopsis thaliana* and *Populus trichocarpa* on callus induction medium [11], [15]. Notably, the genes having an expression peak at 1 h, 3 h, or 6 h could be distinguished, indicating that they are differently regulated with precise mechanisms to be induced or repressed with specific timing.

### Characterization of the Clusters by Gene Ontology Analysis

Our gene ontology (GO) analysis revealed that a large number of genes involved in “photosynthesis-related” GO categories were transiently down-regulated at an early phase, 3 h after dissection. Similar down-regulation of the photosynthesis-related genes was observed in the transdifferentiation process of *Zinnia elegans* mesophyll cells into xylem cells [37] and the callus induction phases of *A. thaliana* [12] and *P. trichocarpa* [15]. Unlike the consistent down-regulation of photosynthesis-related genes in the transdifferentiation and callus induction, the down-regulation during the moss reprogramming is transient and recovers after 12 h. This difference reflects the fact that the xylem cells and callus are usually white with presumably little photosynthetic activity, whereas reprogrammed chloronema cells are green photosynthetic tissue.

Protein kinases were overrepresented in clusters [3,3], which contains genes transiently up-regulated during the reprogramming. These protein kinases could be involved in signal transduction for the reprogramming. Associations between several kinases and wound stress responses have been reported [38] and the protein kinases in the cluster may have similar functions.

GO identifiers for gene expression-related categories were overrepresented in cluster [1], [2], which contains genes up-regulated at later time points, after 6 h. Similarly, among translation-related processes, up-regulation of ribosome-related genes was reported during callus induction in *A. thaliana* and *P. trichocarpa* [12], [15] and during the transdifferentiation of *Z. elegans* [37]. These results are consistent with the idea that global gene expression changes during reprogramming are mediated by regulation of the genes involved in post-transcriptional regulation and translation.

### Expected Roles of Transcription Factors and Epigenetic-related Factors for Reprogramming in *P. patens*

Sequential up-regulation of subsets of *P. patens* transcription factors was observed at specific time points: 1 to 3 h, 3 to 6 h, 6 to 24 h after dissection (Figure 5B). Several transcription factors mediate the pluripotency of ES cells and constitute a transcriptional network [39]. Transcription factors in the moss likely comprise a transcriptional network to facilitate sequential expression of necessary factors including transcription factors and downstream genes for progression of reprogramming.

The *P. patens* reprogramming process is a change of the cellular state from that of differentiated cells to that of stem cells. Chromatin modifications including histone modifications, DNA methylation, and chromatin remodeling affect the nuclear environment for gene expression [35], and epigenetic regulation is employed to maintain the cellular state through a specific gene expression pattern. Cellular states of mammalian stem cells are regulated by these chromatin modifications [40]. Here, we investigated the expression patterns of epigenetic regulation-related factors.

Twenty-six epigenetic regulation-related genes out of 330 epigenetic regulation-related factors (7.9%) were differentially expressed during the reprogramming. We found up-regulation of genes encoding histone modification enzymes (SDGs, HDA, HAM, PRMTs), suggesting that these histone modifications are involved in the reprogramming process. Functional roles for histone modifications in the acquisition of pluripotency were reported in mammalian cells. Tip60-p400 complexes, composed of Tip60 MYST-type histone acetyltransferase and other proteins, have activities for assembly of histone 2A variant (H2A.Z) and histone acetylation, and are required for self-renewal in ES cells [41]. *HAM1502*, encoding a MYST-type histone acetyltransferase in *P. patens*, was up-regulated during the moss reprogramming (Figure 5C). In addition to up-regulation of *HAM1502*, the expression of *H2A.Z* (*HTA1502*) increased. To establish pluripotency, histone acetylation or H2A variant assembly may be employed in moss reprogramming similar to the case of mammal cells. Methylation on histone 4 arginine 3 (H4R3) is mediated by a specific arginine methyltransferase, and symmetric dimethylation (H4R3sme2) regulates flowering and stress responses in *A. thaliana* [42], [43], [44]. PRMT1501 and PRMT1506 are homologues of H4R3 methylase. Methylation on H4R3 was observed in human ES cells [45], indicating that H4R3 methylation may function in pluripotent cells of both human and moss. CHC1503 is one of the SWIB domain-containing SWI/SNF chromatin-remodeling factors. In mammals, a specific ATP-dependent SWI/SNF complex (esBAF) is required for initiation and maintenance of pluripotency [46]. Thus, up-regulation of *CHC1503* suggests that chromatin remodeling also affects the moss reprogramming process.

Non-coding small RNAs are involved in post-transcriptional regulation, translation, and chromatin modifications including DNA methylation and/or histone modification in *A. thaliana* [47]. Small RNAs, miRNA, siRNA and ta-siRNA are produced by several processing factors [47]. Of these, only one double-strand RNA binding protein (DRB) gene was differentially expressed in the 5′-DGE profile, increasing in expression with a maximum at 12 h after dissection (Figure 5C: *DRB1501*). The *A. thaliana* DRB named HYPONASTIC LEAVES 1 (HYL1) was shown to regulate the cleavage of primary miRNA to form specific miRNA species [48]. Specific miRNA species regulate the pluripotency of stem cells in mammals [49], and similar molecular mechanisms for miRNA synthetic pathway may function in *P. patens* reprogramming.

### Auxin and Cytokinin Signaling Likely Function in *P. patens* Reprogramming

The reprogramming of *P. patens* leaf cells into chloronema apical cells does not require exogenous auxin and cytokinin. The expression patterns of auxin and cytokinin metabolism, transport, and signaling genes suggest that these phytohormones are involved in the reprogramming, but are endogenously supplied. A gene encoding a flavin monooxygenase, which is the key enzyme in tryptophan-dependent auxin biosynthesis [30], was induced by 1 h, 3 h and 12 h. *PpGH3-2,* which encodes the enzyme that conjugates IAA with amino acids [31], was induced later than the biosynthetic gene, at 6 h with a peak at 12 h. The up-regulation of an auxin biosynthetic gene is consistent with the idea that intrinsic auxin functions instead of exogenously supplied auxin in *P. patens* reprogramming. The delayed expression of the conjugation enzyme gene could reflect a negative feedback to modulate the auxin level to an adequate concentration. A *RCE1* homolog was transiently up-regulated whereas *SKP* homologs were transiently decreased at 1 h but was induced by 3 h and down-regulated at 6 h. *A. thaliana* RCE1 is an E2 enzyme for RUB [50] conjugation and is involved in auxin receptor complex, SCF^TIR^, assembly with SKP [51], [52], [53]. Induction of these genes may mediate the sensitization to intrinsic auxin.

Cytokinin also has an important role in shoot formation from a callus in flowering plants [4]. *IPT1*, which may function in cytokinin and tRNA biosynthesis, was up-regulated by 6 h. CKX is involved in metabolic degradation of cytokinin [54]. One CKX homolog, *PpCKX7-1*, had biphasic expression pattern; down at 1 h, up at 3 h and down at 6 h. Combined regulation of cytokinin biosynthetic and catabolic enzyme genes is likely to give appropriate concentration of cytokinin for reprogramming. The stem cell system in the shoot apical meristem is also regulated by a cytokinin signal transduction pathway in *A. thaliana* [55], [56]. The phosphotransfer proteins (AHP1 - AHP5) in *A. thaliana* function as positive regulators of cytokinin signaling [57]. Two *P. patens* phosophotransfer protein genes behaved differently during reprogramming. One was transiently repressed, whereas the other was induced by 3 h. The repressed one has a long branch in a phylogenetic analysis (http://moss.nibb.ac.jp/treedb/), implicating a possible functional change during evolution. Similarly, *AHP6,* which has a long branch in the phylogenetic tree, has an inhibitory function [58], in contrast to other phosphotransfer proteins in *A. thaliana*. The induction of the other moss phosophotransfer protein gene may implicate elevated cytokinin sensitivity in later processes of reprogramming. To understand the mechanism of these change in expression levels and the role of these phytohormones in the reprogramming requires further molecular and biochemical studies.

### Conclusion

We established a novel simple 5′-DGE method, which is potentially applicable to profiling of mRNA in any eukaryotic organism. This method has advantages for easy procedure and produces quantitative gene expression data. By using this method, the molecular events of moss reprogramming from leaf cells to chloronema apical cells were described at the transcript level. The analysis revealed distinct gene sets having expression peaks at 1, 3, or 6 h. Further functional analyses of these genes by reverse genetics will shed light for deeper understanding of the reprogramming process.

## Materials and Methods

### Plant Materials and Growth Conditions

Protonemata of *Physcomitrella patens* subsp. *patens* Gransden2004 [9] were homogenized and plated on BCDAT medium solidified with 0.8% agar (WAKO) and grown for 3 to 4 weeks in continuous white light (20–30 µmol/m^2^/s) at 25°C [59]. Upper parts of gametophores with approximately six leaves without rhizoids [60] were cut with scissors and collected in water. For a large scale dissection, the upper parts of gametophores were chopped with a Polytron PT2100 homogenizer with a DA2120/2 generator shaft (Kinematica) for 10 sec at maximum speed two or three times within 1 h after the collection. Dissected gametophores were washed with liquid BCDAT medium twice. Dissected leaves were isolated from stems and cultivated for reprogramming experiments.

### Preparation of 5′-DGE Libraries

The workflow for library preparation is shown in Figure 2. Total RNA was extracted from moss samples using the RNeasy Plant Mini Kit (Qiagen). 5′-DGE libraries were prepared from 5 to 10 µg total RNA. Poly A RNA was enriched with the Fast track kit (Life Technologies). The reverse transcription reaction was performed using PrimeScript II reverse transcriptase (Takara Bio) with biotin-TEG labeled EcoP15I-dT_20_ (BioTEG-dT20EcoP15I) and biotin-TEG labeled-P2-EcoP15I oligonucleotides (BioTEG-P2EcoP15I-rGx3, Table S7). Each reaction consisted of poly A-enriched RNA sample (>100 ng), 1.2 µM BioTEG-dT20EcoP15I and 1.2 µM BioTEG-P2EcoP15I-rGx3 oligonucleotides, 2 mM DTT (dithiothreitol), 0.5 mM dNTP, 1x PrimeScript II buffer, and 1 µl PrimeScript II, in a total reaction volume of 10 µl. After incubation at 42°C for 60 min, alkaline lysis of mRNAs was carried out by the addition of 1 µl 25 mM NaOH and incubation at 68°C for 30 min, then three-cycle PCR was performed with BioTEG-P2EcoP15I-GG and BioTEG-dT20EcoP15I primers to obtain double strand cDNAs. The PCR mix contained 10 µl first-strand cDNA, 0.2 mM dNTP, 1.2 mM MgSO_4_, 0.3 µM BioTEG-P2EcoP15I-GG, 0.3 µM BioTEG-dT20EcoP15I, 1x KOD-Plus buffer, and 2 µl KOD-Plus DNA polymerase (Toyobo), in a total of 100 µl and PCR conditions were one cycle of 95°C for 3 min, 72°C for 10 min, and 95°C for 20 sec, and three cycles of 95°C for 5 sec and 68°C for 8 min. After purification with the MinElute Reaction Cleanup Kit (Qiagen), purified cDNAs were digested with 10 units EcoP15I (New England Biolabs) in a 50 µl reaction volume at 37°C for 16 h. Digested cDNA fragments were captured using the MAGNOTEX-SA kit (Takara Bio) according to the manufacturer’s instructions except for the final step. After washing, the cDNA-attached beads were suspended in 100 µl of 1x T4 DNA ligase buffer (New England Biolabs) for the subsequent ligation step. P1 adaptor with a two-base 5′ overhang to ligate with an EcoP15I fragment was prepared by annealing of P1-A and P1-B-NN oligonucleotides (Table S7). Finally, 100 pmol P1 adaptor was added to the suspension of magnetic beads and ligated to the 3′ ends of cDNA fragments anchored to the beads using 20 units T4 DNA ligase (New England Biolabs) in a 50 µl reaction volume. The ligation mixtures were incubated at room temperature for 2 h, and washed three times with 1x Binding buffer (10 mM Tris-HCl [pH8.0], 1 mM EDTA, 1 M NaCl, 0.1% TritonX-100). Beads were rinsed with 100 µl 1x Phusion HF buffer (Finnzymes) once, and re-suspended in 20 µl 1x Phusion HF buffer for PCR amplification of 5′-DGE libraries. During capture of magnetic beads and the ligation step, 1.5-ml DNA LoBind tubes (Eppendorf) were used. PCR amplification of 5′-DGE libraries carrying P1 and P2 adaptors was performed with high fidelity Phusion DNA polymerase (Finnzymes). Each PCR mix contained 4 µl template bead suspension, 0.2 mM dNTP, 0.5 µM P1 AMP, 0.5 µM P2 AMP, 1× Phusion HF buffer, and 0.5 µl Phusion DNA polymerase in a 50 µl reaction. A total of five reaction tubes were prepared. PCR conditions were 98°C for 30 sec, twelve cycles of 98°C for 10 sec, 65°C for 30 sec, and 72°C for 15 sec, and one cycle of 72°C for 10 min. The amplified 5′-DGE libraries were fractionated by 6% polyacrylamide gel electrophoresis in 1x TBE buffer, and 97 bp DNA fragments were extracted using the QIAEX II Gel Extraction Kit (Qiagen).

### Quantification of SOLiD 5′-DGE Libraries

Precise concentrations of 5′-DGE libraries were determined by real time quantitative PCR (qPCR) with the QuantiTect SYBR Green PCR Kit (Qiagen) on an Applied Biosystems 7500 Real Time PCR System (Life Technologies) with P1-Fw (CCACTACGCCTCCGCTTTCC) and P1-Rv (ATCACCGACTGCCCATAGAG). Ten-fold and fifty-fold diluted libraries were quantified in triplicate using the standard curve method. For a standard curve, serial dilutions of 500, 50, 5, 0.5, and 0.1 pg of reference clone #2 were added to the reactions as templates. Reference clone #2 was established after cloning the test library fragment into a TOPO vector (Life Technologies).

### SOLiD Sequencing

Emulsion PCR (emPCR), bead enrichment, and bead deposition onto slides were performed according to the manufacturer’s protocol (Life Technologies). For 5′-DGE experiments, 25 bp sequencing was routinely conducted. The read data were deposited in DDBJ Sequence Read Archive (DRA) as DRA000400.

### Mapping, Sequenced Tag Assignment and Counting

All scaffolds (a total of 479.99 Mb) of the *P. patens* nuclear genome in the assembly ver. 1.1 [9, ftp://ftp.jgi-psf.org/pub/JGI_data/Physcomitrella_patens/v1.1/Physcomitrella_patens.1_1.fasta.gz], the 0.11 Mb mitochondrial genome [61, NC_007945], and the 0.11 Mb chloroplast genome [62, NC_005087] without one of the inverted repeat regions, were concatenated with 60 Ns after each segment. Thus, a single sequence of 480.33 Mb was prepared as a reference genome. The DNA sequences in color space produced by the SOLiD sequencer were mapped to the concatenated *P. patens* reference genome with no more than 2 mismatches using corona_lite v0.2 (Life Technologies). Reads having 3 or fewer non-0 values were filtered because such reads have little information content and lead to spurious mapping.

A zero-filled array of floating point values with about twice the size of the reference sequence was prepared. The two strands of DNA were distinguished as different positions. For a unique hit read, 1.0 was added to the corresponding position on the array. For a multiple hit read, hits were sorted according to the number of mismatches, the number of locations with the least mismatches (*n*) was counted, and 1/*n* was added to the value in the array corresponding to each location with the least mismatches. The array was written to a binary file for later processing.

From the binary file, the tag counts of a given range could be quickly calculated by summing the numbers corresponding to the region. For visualization as a histogram, a given range was equally divided and the count of every subrange was used to draw a rectangle with the height corresponding to the count (Figure 3A). To extract a gene-wise tag count value, the tag count corresponding to an extended gene model region was calculated. The gene models were extended 2 kb from the 5′ end and 1 kb from the 3′ end in cases where the distance to the neighboring gene model on the same strand is more than 3 kb. For a shorter distance, the intervening region was divided with a 1∶2 ratio. The tag counts were divided by the total number of tags mapped to the genome and multiplied by 10^6^ to obtain the tags per million (TPM) values. These values are available in PHYSCObase at http://moss.nibb.ac.jp/dge/.

### Statistical Analysis of 5′-DGE Tag Data

TPM-based relative tag count data were imported into GeneSpring ver. 7 (Agilent Technologies) for statistical analysis. Per-gene normalization was performed by dividing each time point TPM value with the median. After logarithmic conversion of the normalized value, 1-way ANOVA (parametric test, not assuming equal variance) was performed with a 5% false discovery rate, to extract genes differentially expressed among time points. Normalized time-course data for the differentially expressed genes were used for clustering by the self-organizing map (SOM) method [26]. For gene and condition trees, Spearman correlation and the average linkage algorithm in GeneSpring ver. 7 were used.

### Gene Ontology and Gene-specific Analyses

For GO analysis, a GO annotation file by JGI (ftp://ftp.jgi-psf.org/pub/JGI_data/Physcomitrella_patens/v1.1/Phypa1_1_goinfo_FilteredModels3.tab.gz) was downloaded [9]. We used the BiNGO plugin (http://www.psb.ugent.be/cbd/papers/BiNGO/) for Cytoscape software version 2.6.3 (http://www.cytoscape.org/) [27]. Hypergeometric tests with Benjamini & Hochberg False Discovery Rate (FDR) were carried out using the default parameters. Corrected *p*-values for significantly overrepresented GO terms were obtained. To conduct the gene-specific analyses on transcription factors and epigenetic regulation-related factors, a list of transcription factors was extracted from Plant Transcription Factor Databases v1.0 (http://planttfdb.cbi.pku.edu.cn:9010/download_data/pep/Physcomitrella_patens%28Moss%29.TF.pep), and an epigenetic regulation-related gene list was obtained from ChromoDB (http://www.chromdb.org/). Gene trees were constructed by employing Spearman correlation, and average linkage was used as a clustering algorithm.

### Quantitative RT-PCR

1.0 µg of total RNA treated with DNaseI (Life Technologies) was used for reverse transcription. cDNA was synthesized with the PrimeScript II 1st strand cDNA Synthesis Kit (Takara Bio) using the oligo dT primer. Real time qPCR with the QuantiTect SYBR Green PCR Kit was carried out on an Applied Biosystems 7500 Real-Time PCR system. To evaluate quatitaive nature between qRT-PCR and 5′-DGE, fold change was calculated as following formulas,

Fold change value by qRT-PCR = relative expression level at 3 h/relative expression level at 0 h (relative expression level was determined using *TUA1* gene as the reference as described in [10]).

For comparison between copy number measured by qRT-PCR and 5′-DGE tag data, corresponding EST clones were used as standards to calculate copy number in 0.1 ng polyA RNA. The representative clone and primer sequences are listed in Table S8.

### Selection of Reprogramming-related Orthologous Genes

Reprogramming-related moss orthologues were selected from the database http://moss.nibb.ac.jp/treedb/ [63]. *SHI*, *MYC2*, and *IPT* homologues were selected according to literature [64], [65], [66]. *P. patens* homologs of group IX *ERF* genes were searched by BLASTP using *AT4G17500* as a query.

## Supporting Information

Figure S1 **Comparison TPM values with and without redundantly mapped tags.** TPM value with uniquely mapped tag counts only are plotted against the TPM value calculated by adding the fraction of redundant tags. Genes with less than 0.125 tag are plotted on the position of 0.125 tags.
(TIF)

Figure S2 **Length of transcripts and the TPM values.** (A) The TPM values are plotted against the length of transcripts. (B) The genes were sorted into bins of 1000 genes according to their length and the box plot of TPM values were shown for the bins.
(TIF)

Figure S3 **Correlation of EcoP15I sites and the number of tags.** The number of tags mapped near EcoP15I sites were counted. The cumulative count of each position over all EcoP15I sites on the genome was plotted. The position 0 refers to the first C of the recognition sequence (CAGCAG or CTGCTG).
(TIF)

Figure S4 **Correlation among triplicate 5′-DGE data** Scatter plots of every pair in biological triplicates are shown with Pearson’s correlation coefficient *R* ^2^.
(TIF)

Figure S5 **Expression patterns of** ***PpCYCD;1*** **in 5′-DGE data.** Error bars indicate standard deviation.
(TIF)

Table S1 **Mapping statistics of the SOLiD DGE runs.**
(XLS)

Table S2 **Groups for enriched GO categories in each cluster.**
(XLS)

Table S3 **Kinase in Cluster** [**3,3**] **.**
(XLS)

Table S4 **Homologues of Reprogramming-related genes in** ***Arabidopsis thaliana.***
(XLS)

Table S5 **Clustered transcription factors.**
(XLS)

Table S6 **Clusterd epigenetics-related factors.**
(XLS)

Table S7 **Oligonucleotides used for 5′-DGE.**
(XLS)

Table S8 **Primer sequences used for DGE evaluation.**
(XLS)
