# Supplementary figures and images for: Digital Gene Expression Profiling by 5′-End Sequencing of cDNAs during Reprogramming in the Moss Physcomitrella patens
Source: PLoS One. 2012 May 4;7(5):e36471. doi: 10.1371/journal.pone.0036471 (PMC3344888; doi:10.1371/journal.pone.0036471)

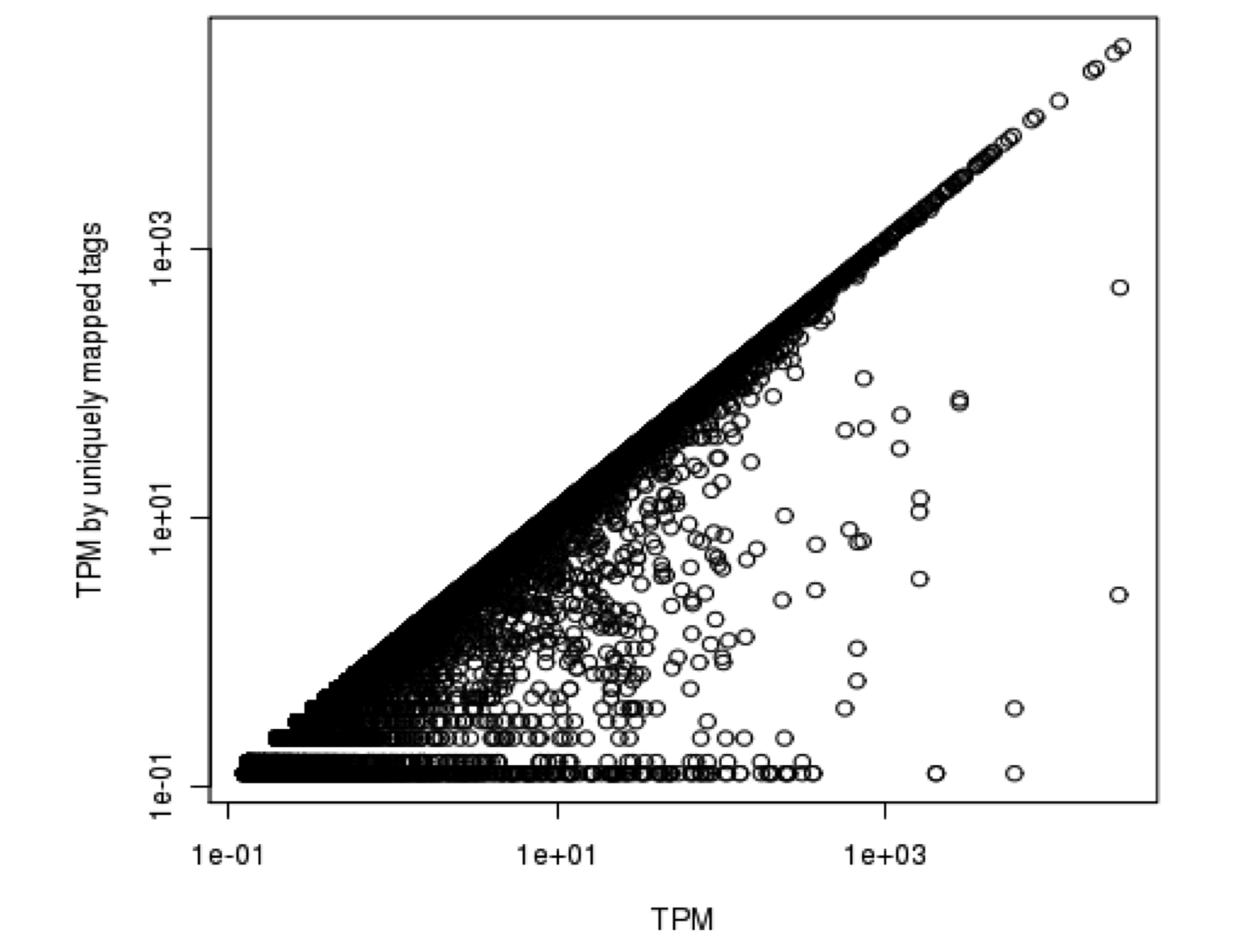

Supplement: Figure S1 — Comparison TPM values with and without redundantly mapped tags. TPM value with uniquely mapped tag counts only are plotted against the TPM value calculated by adding the fraction of redundant tags. Genes with less than 0.125 tag are plotted on the position of 0.125 tags. (TIF) [file pone.0036471.s001.tif]

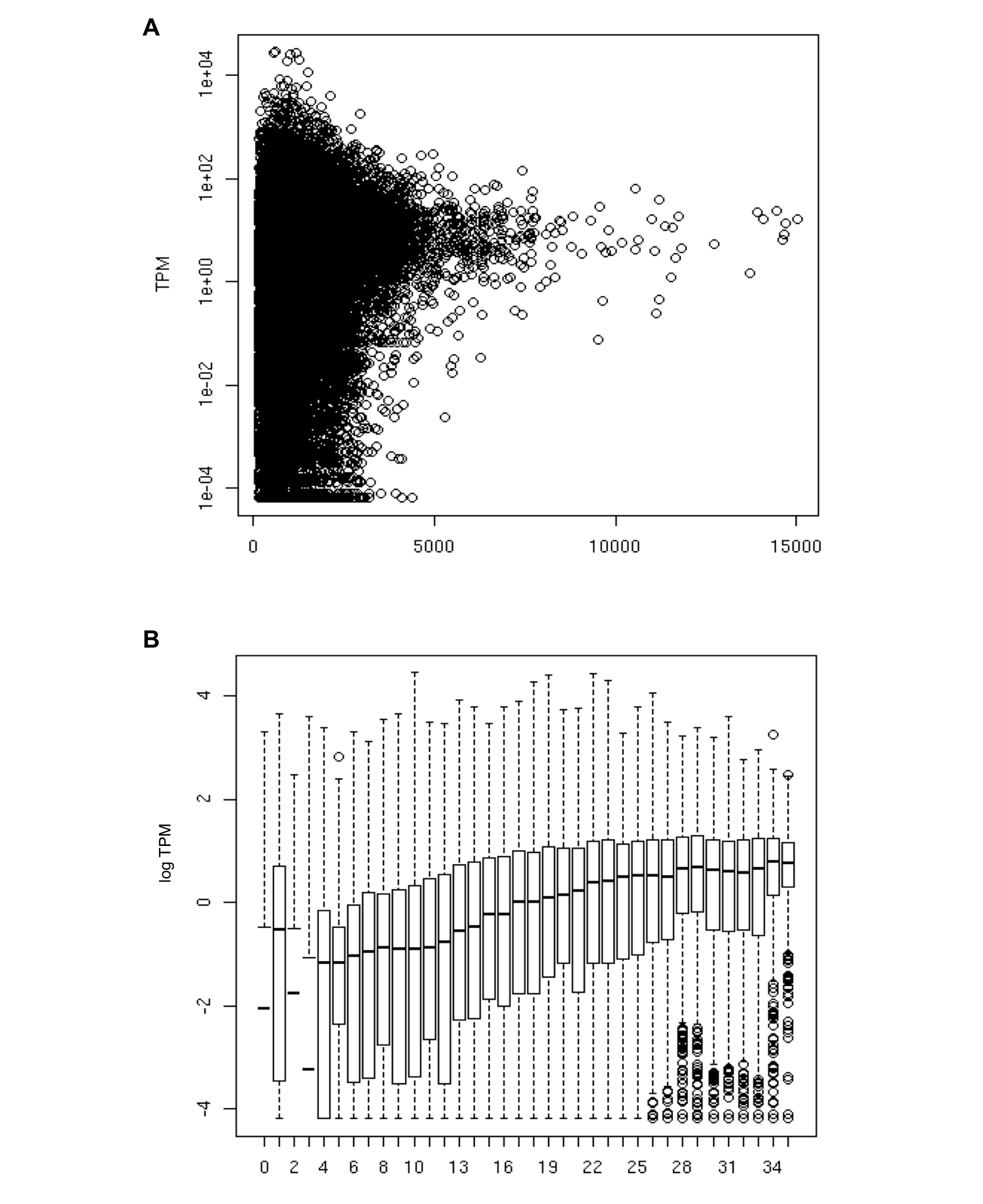

Supplement: Figure S2 — Length of transcripts and the TPM values. (A) The TPM values are plotted against the length of transcripts. (B) The genes were sorted into bins of 1000 genes according to their length and the box plot of TPM values were shown for the bins. (TIF) [file pone.0036471.s002.tif]

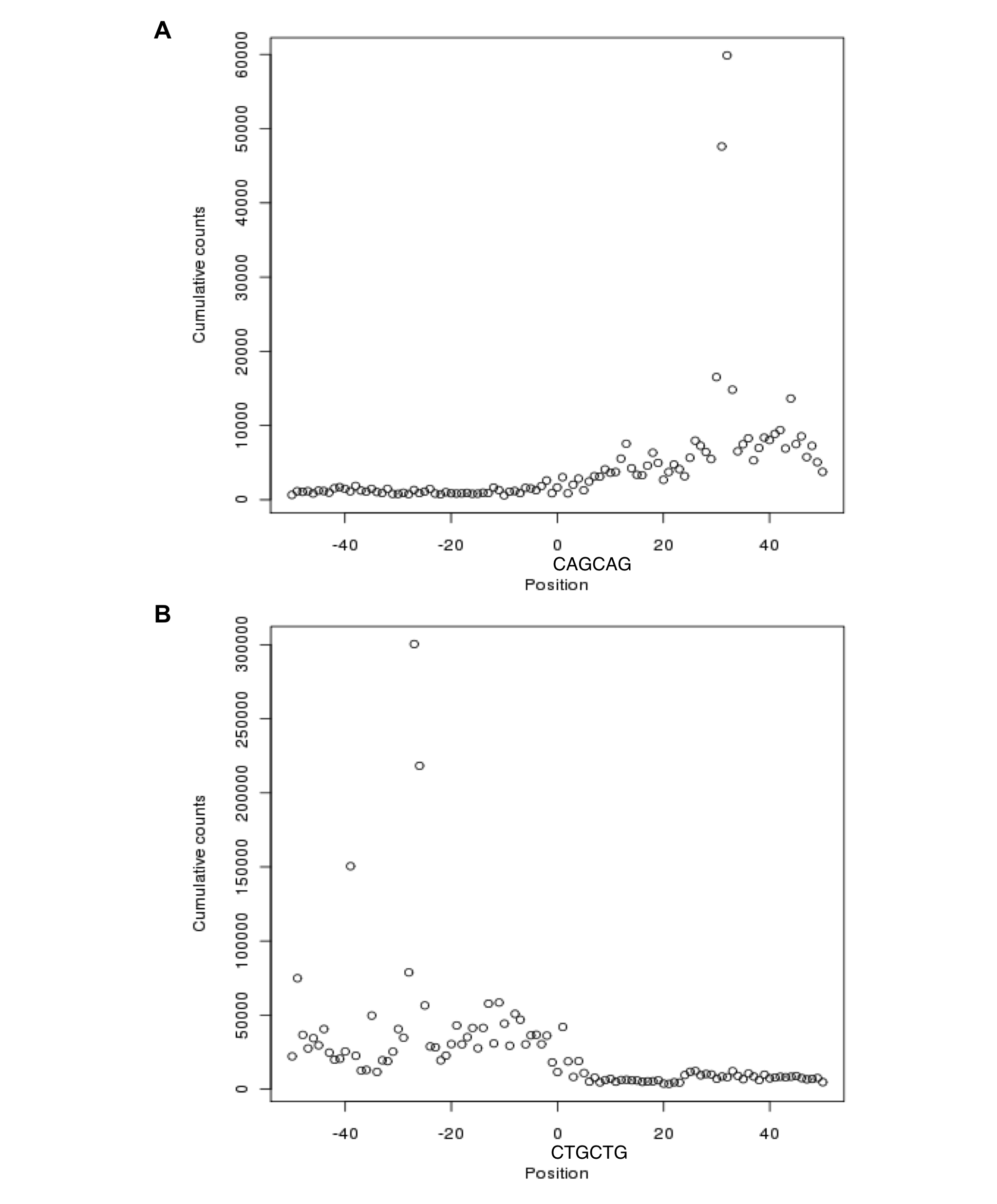

Supplement: Figure S3 — Correlation of EcoP15I sites and the number of tags. The number of tags mapped near EcoP15I sites were counted. The cumulative count of each position over all EcoP15I sites on the genome was plotted. The position 0 refers to the first C of the recognition sequence (CAGCAG or CTGCTG). (TIF) [file pone.0036471.s003.tif]

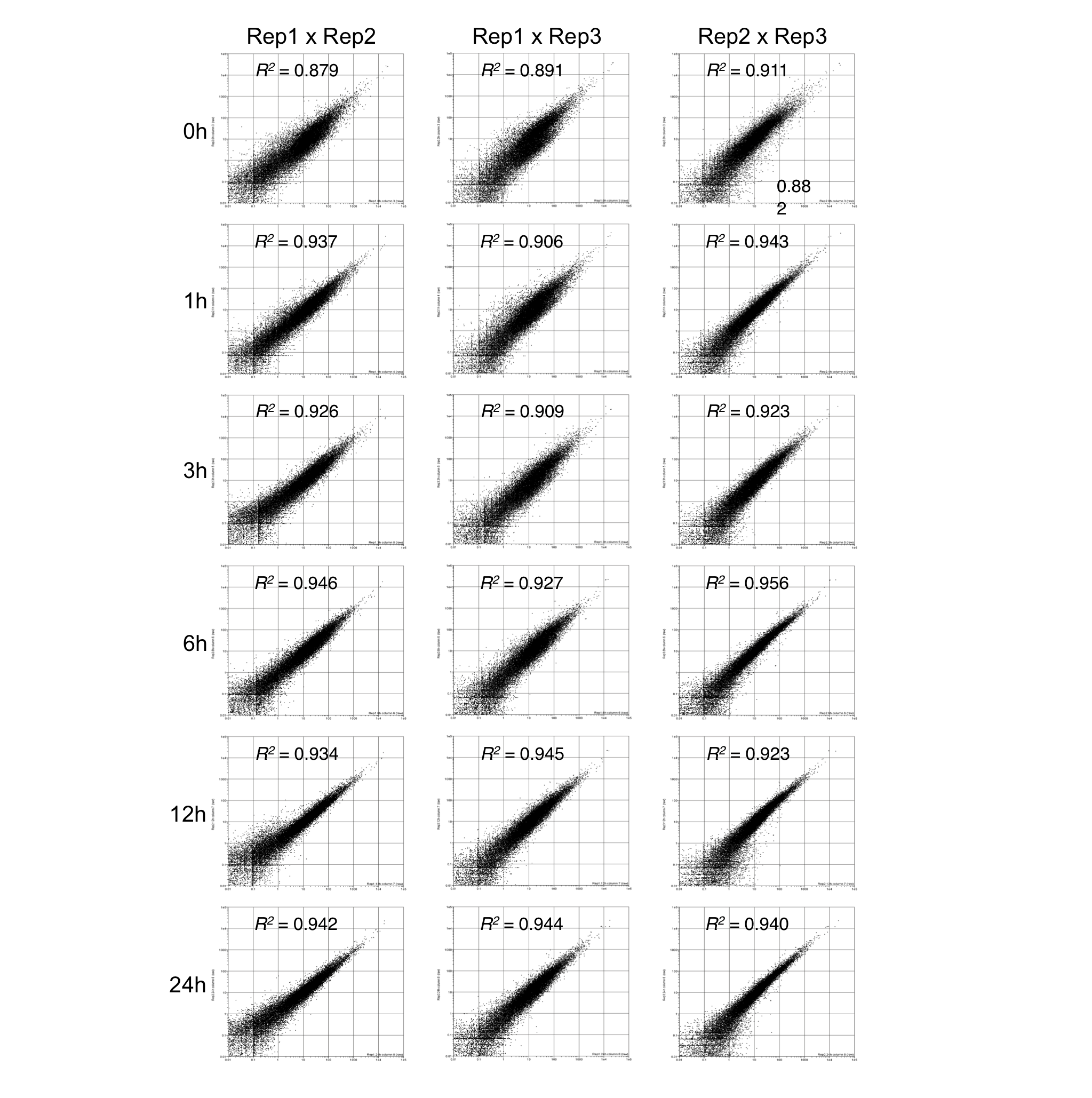

Supplement: Figure S4 — Correlation among triplicate 5′-DGE data Scatter plots of every pair in biological triplicates are shown with Pearson’s correlation coefficient R 2. (TIF) [file pone.0036471.s004.tif]

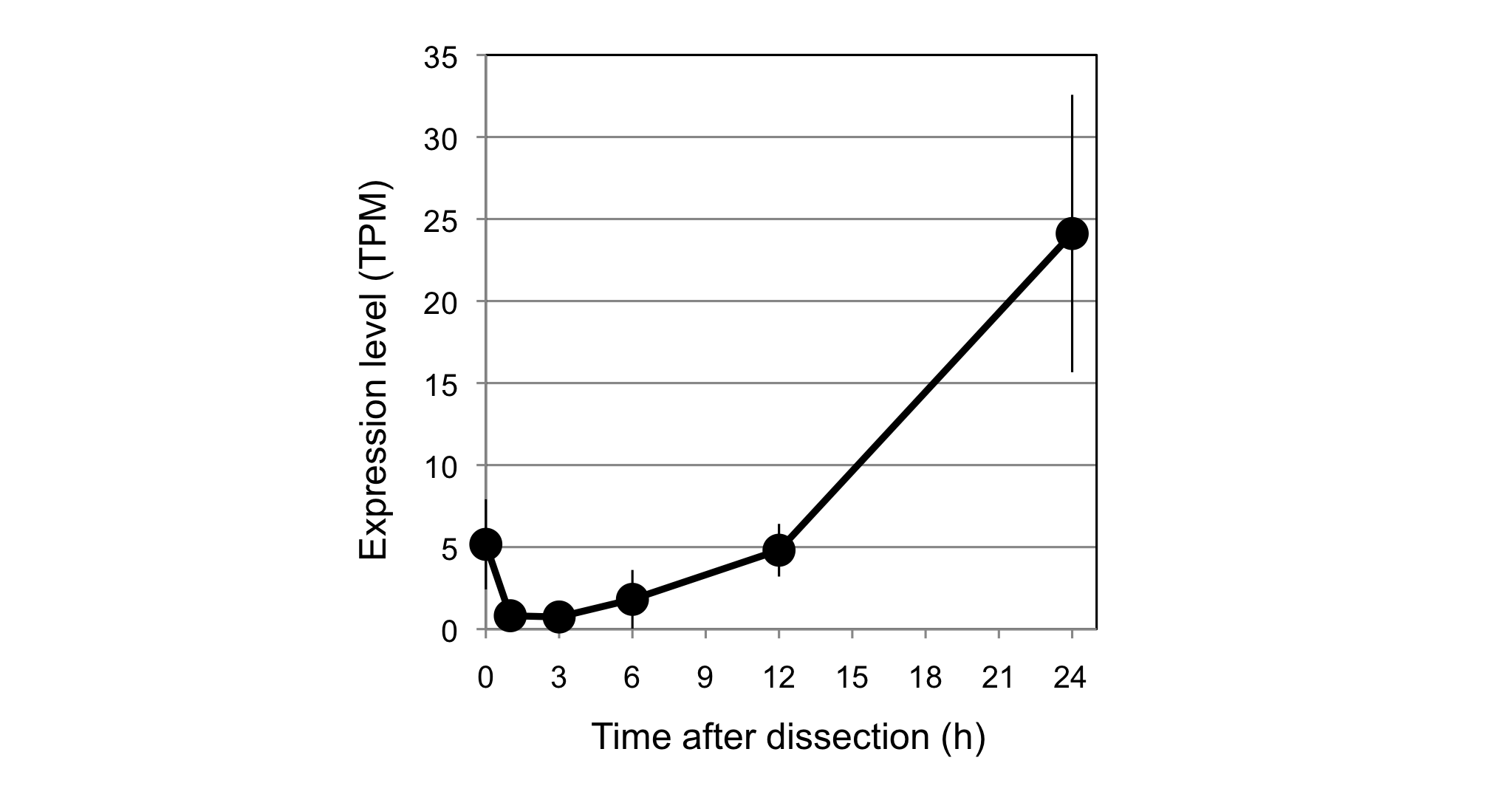

Supplement: Figure S5 — Expression patterns of PpCYCD;1 in 5′-DGE data. Error bars indicate standard deviation. (TIF) [file pone.0036471.s005.tif]
